# A multi-organ atlas of microcirculatory signatures for systemic profiling of diabetic and therapeutic states

**DOI:** 10.1038/s41597-026-07430-w

**Published:** 2026-05-12

**Authors:** Yuan Li, Weiqi Liu, Yingyu Wang, Bing Wang, Xiang Xu, Bingwei Li, Xu Zhang, Mingming Liu

**Affiliations:** 1 Institute of Microcirculation, Chinese Academy of Medical Sciences & Peking Union Medical College, Beijing, 100005 China; 2 International Center of Microvascular Medicine, Chinese Academy of Medical Sciences, Beijing, 100005 China; 3 Diabetes Research Center, Chinese Academy of Medical Sciences, Beijing, 100005 China; 4 Laboratory of Electron Microscopy, Ultrastructural Pathology Center, Peking University First Hospital, Beijing, 100034 China

**Keywords:** Diabetes complications, Pre-diabetes

## Abstract

Microcirculatory deterioration in diabetes mellitus causes severe organ-specific complications, yet a systemic understanding of its cross-organ pathophysiology remains elusive due to a lack of comprehensive data. To address this gap, we present a high-dimensional dataset mapping microhemodynamic and oxygenation profiles across six organs in murine models of health, pre-diabetes, and type 1 and 2 diabetes. Structured as a third-order tensor, the dataset comprises 10-parameter physio-signatures for each condition, documenting responses to insulin and the GLP-1 receptor agonist liraglutide at one- and two-week endpoints. Our resource enables direct deconvolution of disease- and organ-specific signatures and provides a quantitative platform for comparing therapeutic pharmacodynamics. We propose a vectorial and tensorial analytical framework to dissect systemic patterns, quantify disease perturbation, and identify significant drug-organ interactions. Our foundational dataset is intended to catalyze the development of system-level computational models for managing diabetic microvascular disease.

## Background & Summary

The microcirculation functions as the principal site of material exchange between blood and tissue^1–3^, playing an indispensable role in maintaining organ-specific metabolic function and structural integrity^4–6^. In metabolic disorders such as diabetes mellitus, the microvasculature undergoes deleterious remodeling^7–9^. The pathophysiology is multifaceted, involving basement membrane thickening^10,11^, altered vasomotion^12^, increased vascular permeability^13,14^, and impaired rheological properties of blood cells^15–17^.

While the systemic nature of diabetes is well-recognized, research into its microvascular consequences often proceeds in an organ-centric manner. Furthermore, existing public datasets predominantly focus on clinical outcomes or omics data^18,19^, leaving an absence of datasets that systematically map multi-parameter hemodynamic and oxygenation profiles across multiple organs and disease stages^20,21^. Consequently, computational models of microvascular function remain algorithm-rich but data-poor^22–24^, as simulations of blood rheology and oxygen transport^25–28^ lack the multi-organ empirical data required for systemic calibration.

To address the gap, we present a dataset characterizing microcirculatory profiles across six distinct organs (colon, intestine, kidney, pancreas, skin, testis) in murine models. The dataset captures the microvascular pathological states driven by absolute insulin deficiency (type 1 diabetes, T1D), systemic insulin resistance (type 2 diabetes, T2D), and a high-fat diet (HFD) pre-diabetic model. Additionally, it documents the microvascular response to insulin, known to affect vasodilation^29–31^, and the GLP-1 receptor agonist liraglutide, which has reported pleiotropic vascular benefits^32–34^, at 1- and 2-week endpoints.

For each experimental condition, the dataset provides a 10-dimensional physio-signature vector. Tissue oxygenation metrics include RBC tissue fraction (C_RBC_), oxygen saturation (SO_2_), and hemoglobin concentrations. The microhemodynamic profiles consist of total blood perfusion and a velocity-stratified analysis (low-speed, mid-speed, and high-speed components), which is critical for distinguishing nutritive capillary perfusion from metabolically inefficient arteriovenous shunting. Our multi-organ data resource serves as a foundational empirical asset for dissecting disease signatures, evaluating therapeutic responses, and providing boundary conditions for multi-scale computational models of systemic microvascular failure.

## Methods

### Ethical approval and animal husbandry

All procedures were approved by the Institutional Animal Care and Use Committee of the Institute of Microcirculation, CAMS (Approval No. CAMS-IM-IACUC-AE0377). 8 weeks old male BALB/c mice were housed in a specific-pathogen-free facility (12:12 h light-dark cycle, 26 ± 1 °C, 55–70% humidity) with ad libitum access to standard chow and water.

### Induction of murine models of metabolic disease

The BALB/c genetic background was selected for disease modeling due to its well-documented stability in baseline microvascular architecture^35,36^. By employing a strain exhibiting relative resistance to spontaneous metabolic deterioration, the experimental design is structured to attribute subsequent multiparametric microcirculatory perturbations specifically to the defined chemical and dietary interventions, thereby reducing background variability in baseline reference vectors.

**Fig. 1:**
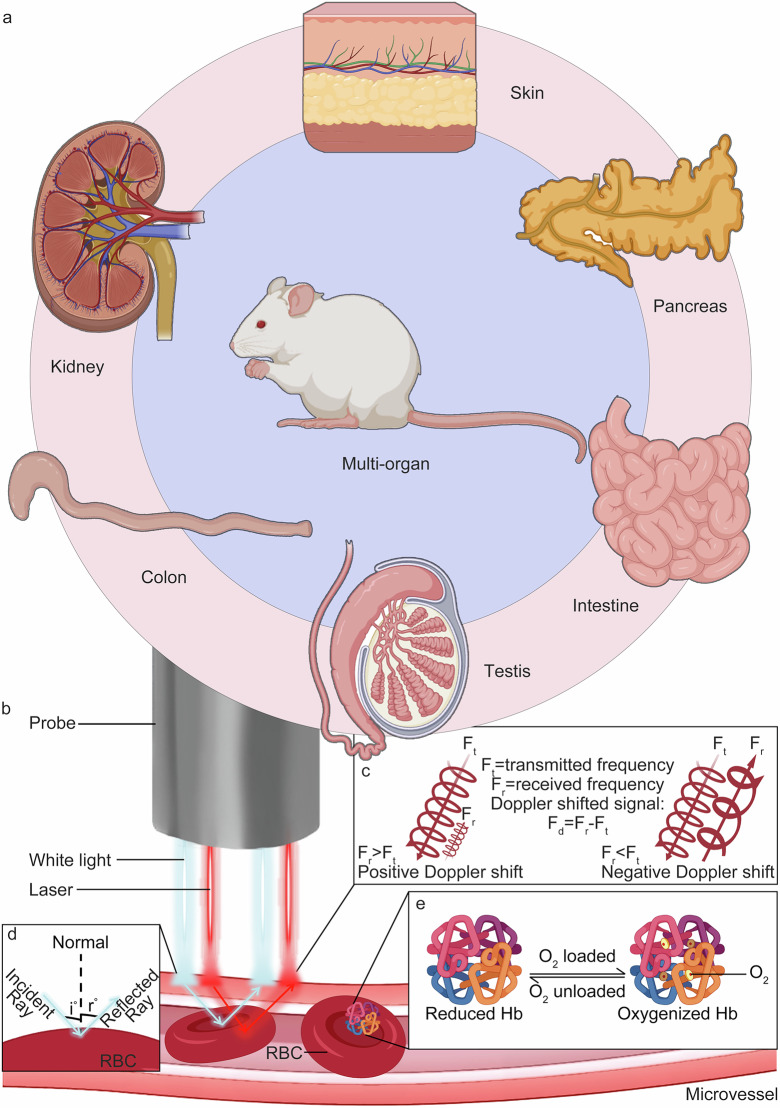
Schematic of the multi-organ microcirculatory assessment platform. (**a**) Systematic profiling of the microcirculation across six harvested organs (skin, pancreas, intestine, testis, colon, and kidney). (**b**) A fiber-optic probe integrating laser Doppler flowmetry and diffuse reflectance spectroscopy was used for non-invasive, real-time monitoring of microhemodynamics and oxygenation. (**c**) Principle of laser doppler flowmetry. The schematic illustrates the use of the Doppler effect to quantify microcirculatory blood flow, where the frequency shift (*Fd* = *Fr*-*Ft*) of back-scattered light from moving RBCs is proportional to their velocity. (**d,e**) Principle of DRS for optical assessment of hemoglobin oxygenation. The spectrum of the diffusely reflected light is analyzed to determine the relative concentrations of oxygenated and reduced hemoglobin based on distinct light absorption profiles. The diffusely reflected light spectrum is analyzed to quantify oxygenated and deoxygenated (reduced) Hb based on different absorption profiles. RBC, red blood cell; Hb, hemoglobin.

Following acclimatization, a total of 180 mice were enrolled in the study and distributed across 12 experimental groups (*n* = 15 per group). The animals were initially allocated into a Control group (*n* = 15), a T1D cohort (*n* = 75), and an HFD/T2D cohort (*n* = 90) (Fig. 1 and Supplementary Fig. 1). The pre-diabetic (HFD) model was established by feeding the HFD/T2D cohort a 60% high-fat diet (kcal %; Cat. No. D12492; HFK Bioscience, Beijing, China) for 4 weeks. Subsequently, 15 of these mice were maintained as the HFD baseline group, while the remaining 75 mice were subjected to low-dose STZ injections (Sigma-Aldrich, Darmstadt, Germany; 50 mg/kg in 0.1 M citrate buffer, pH 4.3) on two consecutive days to generate the T2D model. The T1D model was induced via a single intraperitoneal (IP) injection of STZ (Sigma-Aldrich; 150 mg/kg body weight), freshly dissolved in 0.1 M citrate buffer (pH 4.3). Fasting blood glucose (FBG) was assessed from tail vein samples using a One Touch UltraEasy® glucometer (Johnson & Johnson, CA, USA). Both types of diabetes were confirmed by an FBG > 200 mg/dL. Ultimately, the T1D and T2D cohorts each yielded 75 successfully modeled mice (15 for the untreated disease baseline and 60 for the subsequent therapeutic arms). Due to refined modeling protocols, all enrolled animals successfully met the inclusion criteria, and no animal mortality or unexpected attrition occurred during the study period. The detailed sample sizes and evaluated endpoints for each group are summarized in Supplementary Table 1.

### Therapeutic intervention protocol

Confirmed T1D (*n* = 75) and T2D (*n* = 75) mice were randomized into three daily IP treatment arms: vehicle control (saline), insulin (1 IU Humalog® Mix25; Eli Lilly, IN, USA), and liraglutide (0.2 mg/kg; Novo Nordisk, Copenhagen, Denmark). Outcome data were collected at two predefined time points, one week and two weeks following the initiation of treatment.

### Microcirculatory data acquisition and analysis

Under 2% isoflurane anesthesia and strict thermoregulation (37.0 ± 0.5 °C) using heating pads to stabilize core body temperature and minimize temperature-induced vascular fluctuations, target organs were sequentially exposed and kept moist with pre-warmed saline. Microhemodynamics and oxygenation were quantified using an integrated laser Doppler flowmetry and diffuse reflectance spectroscopy system (PeriFlux 6000 with EPOS software; Perimed AB, Stockholm, Sweden). A fiber-optic probe was positioned perpendicularly on the surface of each organ. Data were recorded continuously for 60 secs from a representative tissue site, yielding 10 real-time optical parameters (Table 1), including conventional perfusion (PU), total blood perfusion (%RBC × mm/s), and speed-resolved perfusion profiles. Concurrently, the DRS component provided tissue oxygenation metrics, including the red blood cell tissue fraction (C_RBC_, %), oxygen saturation (SO_2_, %), total hemoglobin concentration (Total Hb, µM), oxygenated hemoglobin concentration (oxygenized-Hb, µM), and reduced hemoglobin concentration (reduced-Hb, µM).

**Table 1:** Data dictionary of microcirculatory oxygen and microhemodynamic parameters.

Variable name	Description	Unit
C_RBC_	Fraction of the sampling volume that consists of RBCs	%
Total Hb	Amount of Hb in the sampling volume	μM
Oxygenized Hb	Total Hb × SO_2_	μM
Reduced Hb	Total Hb × (1 - SO_2_)	μM
SO_2_	C_RBC_ or Hb in the sampling volume that is saturated	%
Total perfusion	C_RBC_ × average speed	% RBC × mm/s
Low-speed perfusion	Blood perfusion with whose speed below 1 mm/s	% RBC × mm/s
Mid-speed perfusion	Blood perfusion with whose speed at 1–10 mm/s	% RBC × mm/s
High-speed perfusion	Blood perfusion with whose speed above 10 mm/s	% RBC × mm/s
Conventional perfusion	Relative blood perfusion	PU

Note: RBC, red blood cell; C_RBC_, red blood cell tissue fraction; Hb, hemoglobin concentration; SO_2_, oxygen saturation.

### Data preprocessing and outlier handling

Data preprocessing was performed in Microsoft Excel (v. 16.100.1; Microsoft Corp., Redmond, WA, USA). Outliers for each parameter were identified using the standard interquartile range (IQR) method (values outside Q_1_ – 1.5 × IQR and Q_3_ + 1.5 × IQR) and were subsequently addressed using automated winsorization.

### Data visualization and functional profiling

**Fig. 2:**
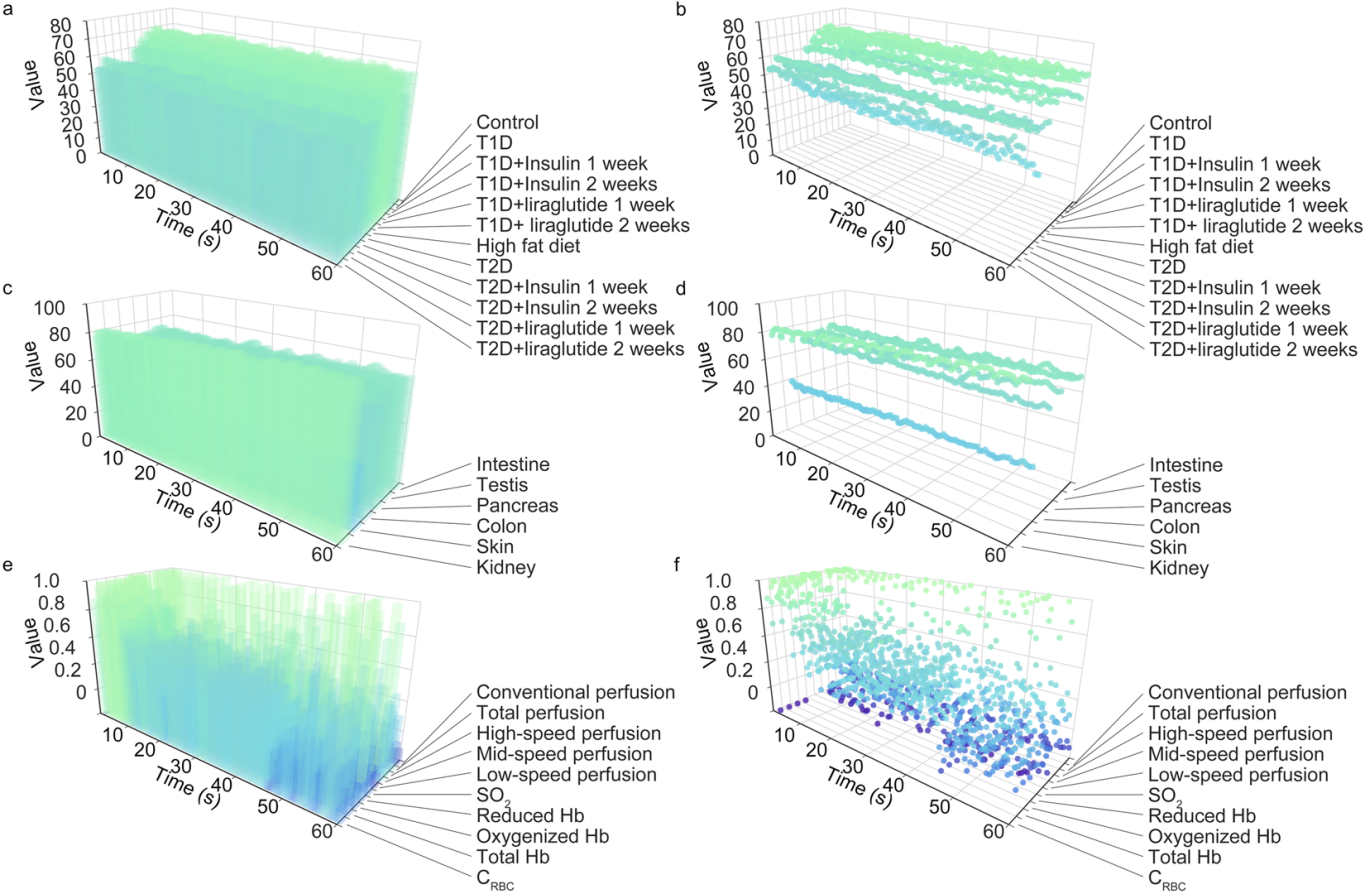
Three-dimensional visualization of the third-order tensor data structure. The multi-axial nature of the dataset is illustrated by slicing the data tensor along its three primary dimensions, experimental state, organ, and physiological parameter. The left column (**a,c,e**) displays data as continuous surface plots, while the right column (**b,d,f**) presents discrete scatter plots. In all panels, the X-axis represents the 60 sec measurement time, and the Z-axis represents the parameter’s value. (**a,b**) Intervention response vector (W_jk_), tracing the trajectory of a single parameter across all 12 experimental states (Y-axis), revealing the systemic impact of disease and therapy on that specific metric. (**c,d**) Organ distribution vector (U_ik_), illustrating the value of a single parameter across all six organs under a fixed condition. (**e,f**) Physio-signature vector (V_ij_), showing the complete profile of all 10 measured parameters for a single condition. Z-axis are transformed using Min-Max normalization to a dimensionless range of [0, 1], as indicated by the color bar. C_RBC_, red blood cell tissue fraction; SO_2_, oxygen saturation; Hb, hemoglobin concentration; T1D, type 1 diabetes; T2D, type 2 diabetes.

Line charts illustrate parameter means and 95% confidence intervals were generated using Python (v. 3.7.4; Python Software Foundation, www.python.org). Reporting 95% confidence intervals provides an estimate of the precision of the multi-parameter microcirculatory profiles across experimental groups. To visualize the holistic microcirculatory state, composite 3D functional plots were created using the Apache ECharts library (v. 6.0.0; Apache Software Foundation) (echarts.apache.org). Disparate variables were integrated into a single coordinate system using Min-Max normalization, x’ = (x – min)/(max – min), scaling all parameters to a common dimensionless range to facilitate simultaneous cross-parameter comparisons (Fig. 2).

## Data Record

**Fig. 3:**
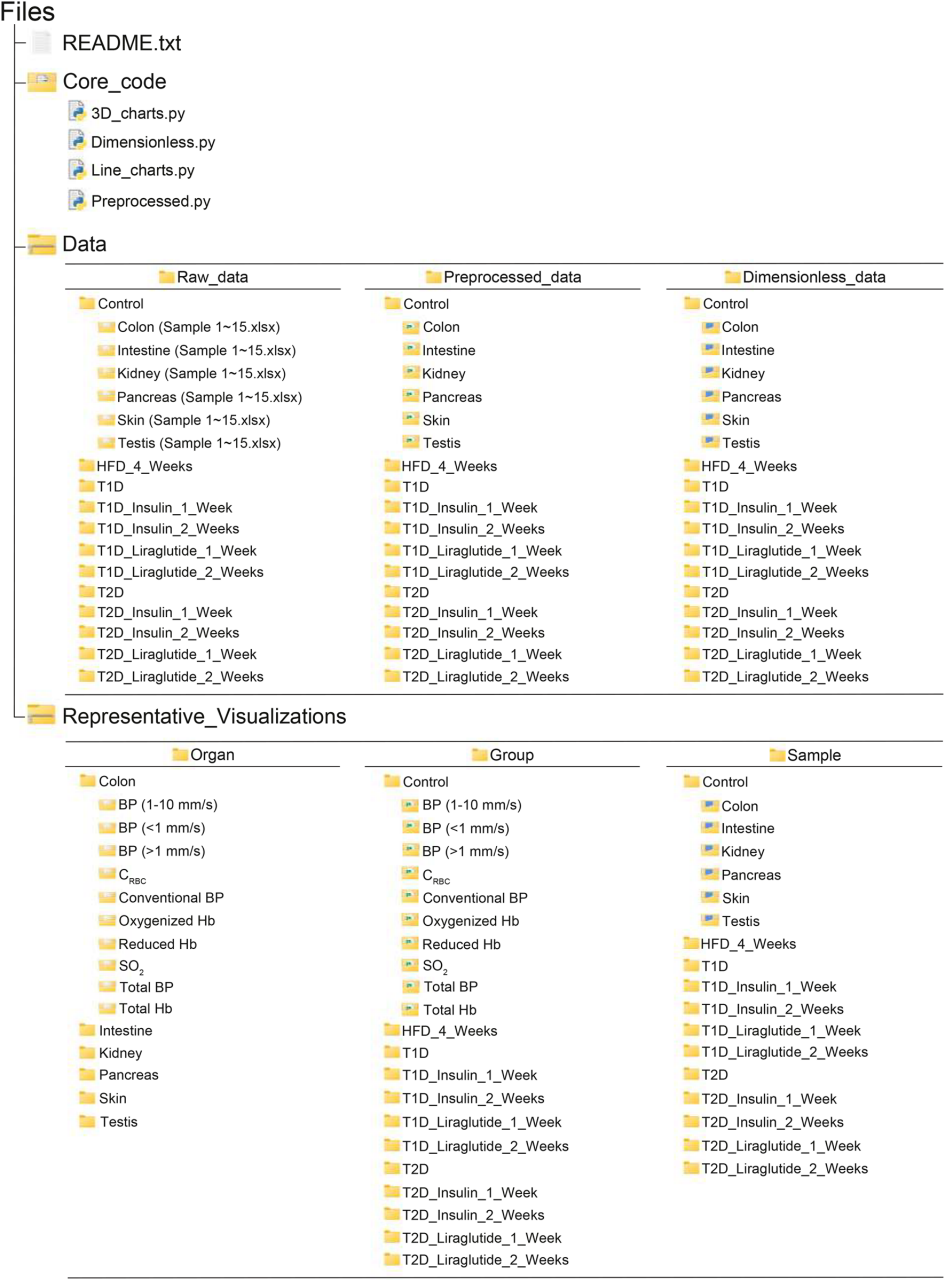
Hierarchical architecture of the project repository. The directory structure comprises four primary modules. The *README* module provides project documentation and usage guidance. The *Core_code* module contains scripts for multi-scale data processing and visualization. The Data module archives raw, preprocessed, and dimensionless datasets that are systematically organized according to experimental conditions and organ types. The *Representative_Visualizations* module stores the generated graphical outputs along with their associated rendering dependencies.

The complete dataset comprises continuous 60 sec recordings from 180 mice (15 animals per 12 experimental groups) with no missing data points (Fig. 3, Supplementary Fig. 2)^37^. The repository is structured hierarchically that the root directory contains a *README.txt* file detailing variable definitions. The *Raw_Data* directory is partitioned by experimental group (e.g., Control, T1D_Insulin_1w), and further subdivided by the six investigated organs. Individual animal data are stored in *Microsoft Excel files (.xlsx)*, where rows represent time points and columns represent the 10 quantified microcirculatory parameters. A supplementary *Representative_Visualizations* folder provides illustrative line charts (.png) and interactive 3D functional diagrams (.html) generated from the primary data.

## Technical Validation and Analytical Framework

### Data quality control and signal integrity

To ensure the high fidelity of the microcirculatory dataset, quality control (QC) protocols were implemented during both the data acquisition and processing phases. Prior to *in vivo* measurements, the imaging system was calibrated to ensure baseline stability. During data extraction, optical microcirculatory signals were continuously monitored for signal-to-noise ratio (SNR) and motion artifacts. Any data epochs exhibiting baseline drift or motion-induced signal corruption were systematically flagged and excluded from the final quantitative analysis automatically. The screening guarantees that the extracted microcirculatory parameters reflect physiological states rather than technical noise.

### Demonstration of practical interpretability and data utility

To demonstrate the practical interpretability and analytical potential of multi-axial dataset, we performed a Principal Component Analysis (PCA) as a representative example of downstream data mining. By treating the 12 × 6 = 72 experimental conditions as samples and the 10 microcirculatory parameters as features, we formed a data matrix to explore the dominant modes of systemic variation across the entire dataset.

**Fig. 4:**
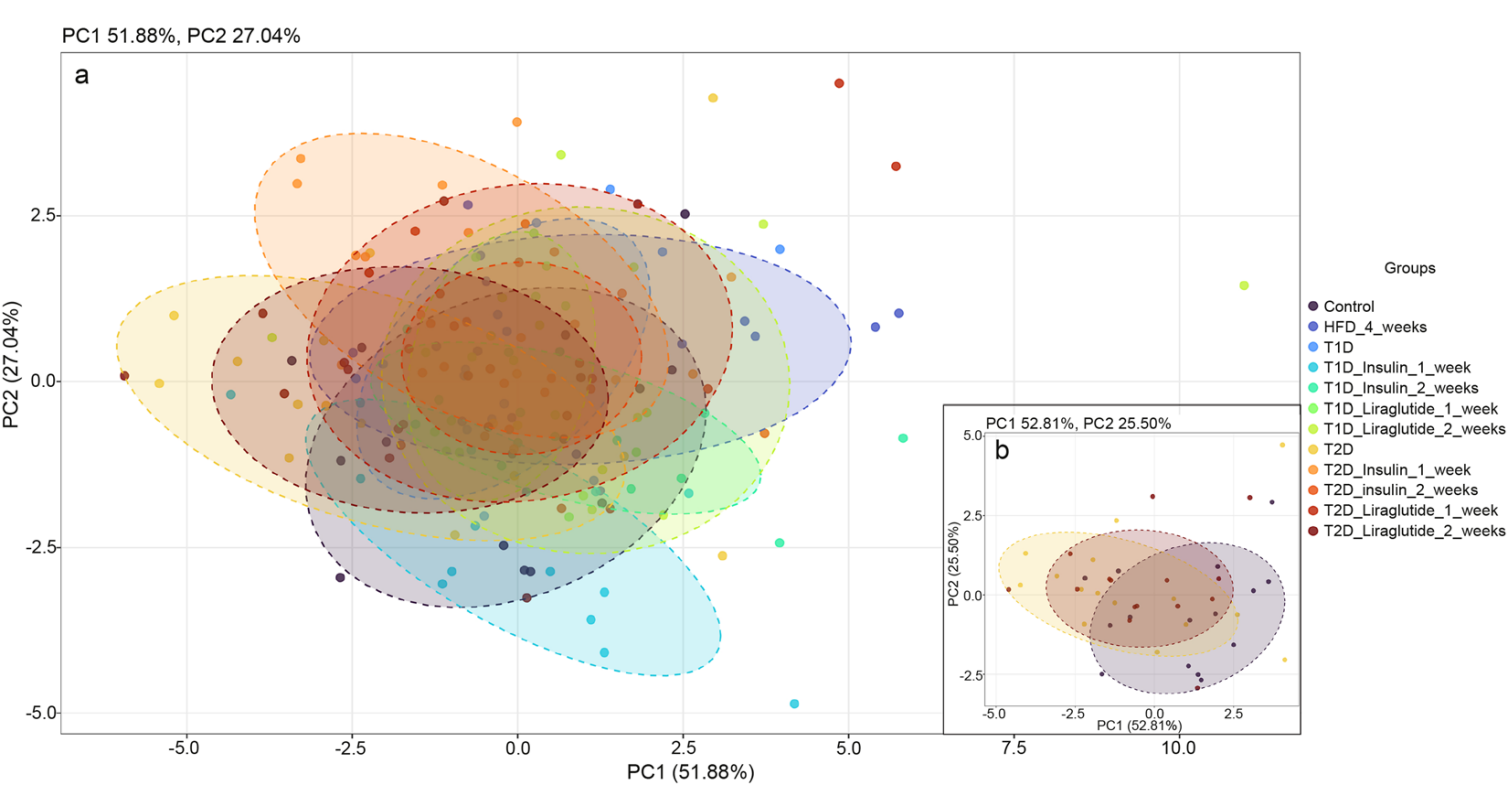
Quality control of preprocessed data. (**a**) PCA score plot illustrating the clustering of 15 samples across all 12 groups based on 10 microcirculatory parameters. (**b**) PCA score plot of 3 representative groups, including healthy (control), disease (T2D), and treatment (T2D_Liraglutide_2_Weeks) groups. Colored dots represent individual samples. PCA, principal component analysis; HFD, high fat diet; T1D, type 1 diabetes; T2D, type 2 diabetes.

As shown in Fig. 4, the PCA projection synthesizes the high-dimensional data into a visually interpretable lower-dimensional space, with the first two principal components (PC1 and PC2) capturing a substantial portion of the total variance (51.88% and 27.04%, respectively). Despite biological variability, the visualization reveals clustering patterns and observable spatial shifts between the healthy control group, the respective disease models, and the post-treatment groups. The distribution facilitates both qualitative interpretation and quantitative evaluation of disease progression and therapeutic effects.

Furthermore, to provide a more detailed perspective on mechanistic recovery, an inset plot focusing on a targeted subset of experimental groups is included (Fig. 4, inset: PC1 52.81%, PC2 25.50%), that highlighting clearer vector-similarity comparisons and demonstrating how researchers can quantitatively assess the degree to which a specific pharmacological intervention restores the microcirculatory profile toward the baseline state.

In addition to spatial clustering, the loadings of each principal component (i.e., the coefficients representing the linear combinations of the original 10 microcirculatory parameters) reveal underlying biological meanings. For instance, a PC heavily loaded with low-speed, mid-speed, and total perfusion can be interpreted as a general perfusion axis. By employing multivariate approaches, researchers can uncover complex, cross-parameter relationships that might not be apparent through univariate analysis.

**Fig. 5:**
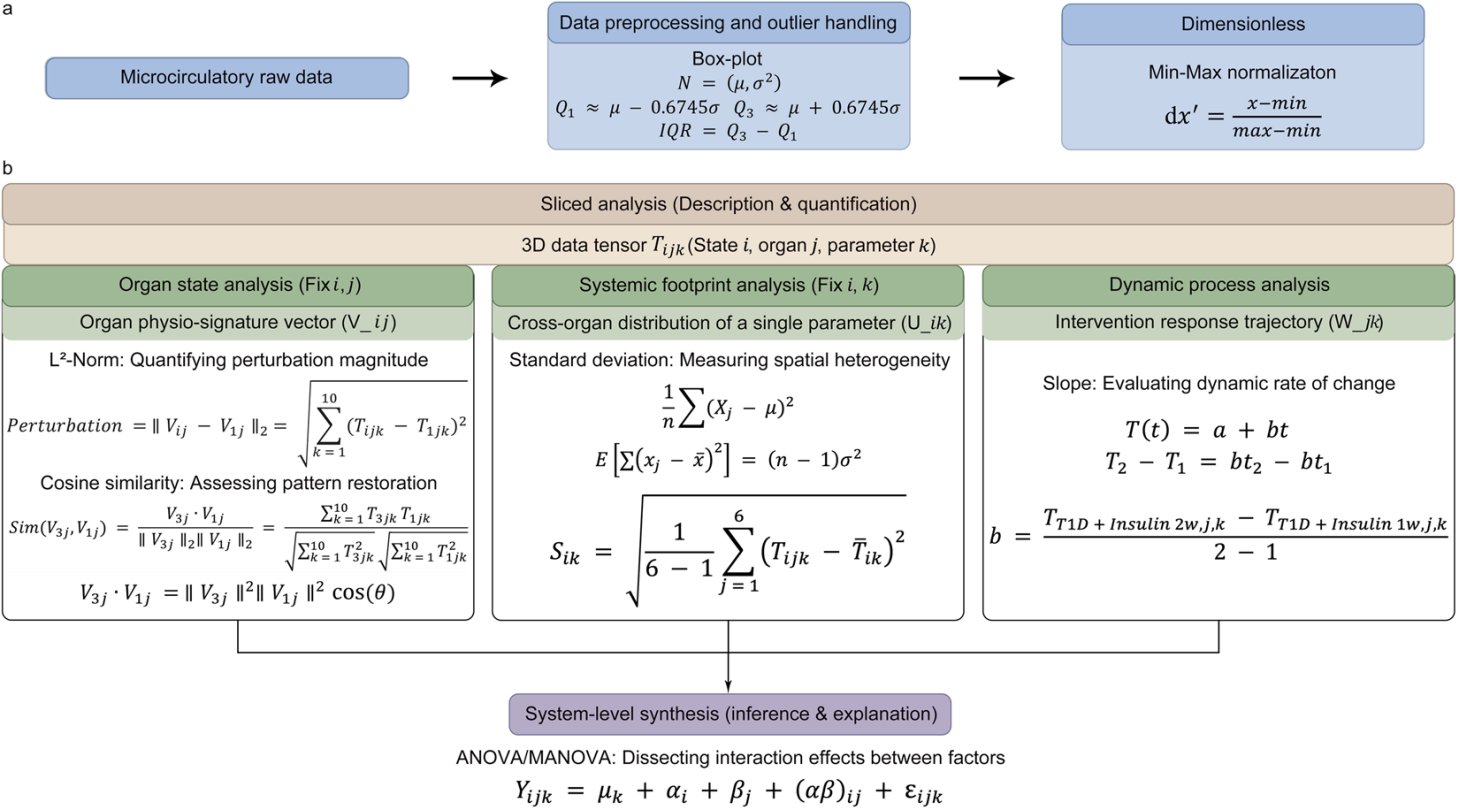
Schematic of the multi-scale data processing and analytical framework. A pipeline for transforming raw microcirculatory data into quantitative insights and system-level models. (**a**) Data preprocessing workflow. Raw time-series data undergo outlier management via winsorization (1.5 × IQR) and are rendered dimensionless using Min-Max normalization for cross parameter comparison. (**b**) Core analytical framework. The dataset is structured as a third-order tensor, *T*_*ijk*_, with axes representing experimental state (*i*), organ (*j*), and parameter (*k*). A sliced analysis approach deconstructs the tensor into three different vectors. Organ physio-signature vector (*V*_*ij*_) for condition-specific profiles. The magnitude of its perturbation from a baseline is quantified using the Euclidean norm (L²-norm), while the restoration of its physiological pattern following therapy is assessed using cosine similarity. Organ distribution vector (*U*_*ik*_) for quantifying organ-specific heterogeneity. Standard deviation (*S*_*ik*_) is calculated as a quantitative index of organ-specific heterogeneity in response to a given condition. Intervention response vector (*W*_*jk*_) for tracing pharmacodynamic trajectories. The slope (*b*) between therapeutic time points is used to estimate the dynamic rate of change. The framework culminates in system-level synthesis, where multi-way statistical models like ANOVA or MANOVA are employed to dissect the main effects of interventions (*α*_*i*_), main effects of organs (*β*_*j*_), and the interaction effects between them *(αβ)*_*ij*_.

In summary, our dataset represents a quality-controlled repository of microcirculatory measurements that is highly amenable to multi-scale analytical frameworks. A mathematical framework for further derivative analyses (Fig. 5), including tensor-based vectorial representation, organ heterogeneity indexing, and multi-way ANOVA modeling, is provided in the Supplementary Materials.

### Limitations

A primary limitation of the current dataset is the reliance on a single murine genetic background. Because BALB/c mice exhibit a comparatively mild metabolic phenotype, the magnitude of the calculated perturbation vectors may not fully capture the microvascular deterioration observed in metabolically susceptible models. Future investigations should incorporate diverse genetic backgrounds to quantify genetic variability and validate the generalizability of the proposed tensorial analytical framework.

## Supplementary information

A multi-organ atlas of microcirculatory signatures for systemic profiling of diabetic and therapeutic states

## Data Availability

The datasets generated and analyzed are publicly accessible in the ZENODO repository^37^ under the digital object identifier 10.5281/zenodo.17197710.

## Code availability

All custom code developed for the processing, analysis, and visualization of the data presented in this manuscript is publicly available^37^. The repository includes Python (version 3.7.4; Python Software, DE, USA) scripts for:

Processing of raw laser signals and hyperspectral data to calculate the 10-parameter physio-signature vectors, including RBC tissue fraction (C_RBC_), SO_2_, and hemoglobin concentrations (Hb). Implementation of the velocity-stratified perfusion algorithm to separate total perfusion into low-, mid-, and high-speed components. Statistical analyses presented in the manuscript, including the scripts used for modeling main and interaction effects between experimental groups and organs. Generation of all figures and statistical plots. The repository contains a detailed *README*.txt file with instructions for execution, a list of required toolboxes, and sample data to ensure reproducibility. Data acquisition was performed using the proprietary EPOS Software named EPOS Manager. This commercial software is not part of the provided code repository and is governed by its own licensing terms.
