# Supplementary material for: A multi-organ atlas of microcirculatory signatures for systemic profiling of diabetic and therapeutic states
Source: Sci Data. 2026 May 12;13:1117. doi: 10.1038/s41597-026-07430-w (PMC13427728; doi:10.1038/s41597-026-07430-w)
Supplement: Supplementary file 1 — A multi-organ atlas of microcirculatory signatures for systemic profiling of diabetic and therapeutic states [file 41597_2026_7430_MOESM1_ESM.pdf]

Supplementary Materials

A multi-organ atlas of microcirculatory signatures for systemic profiling of diabetic and therapeutic states

Yuan Li[1,2,3#], Weiqi Liu[1,2#], Yinguyu Wang[1,2], Bing Wang[1,2,3], Xiang Xu[1,2], Bingwei Li[1,2]
Xu Zhang[4], Mingming Liu[1,2 3]*

[1] Institute of Microcirculation, Chinese Academy of Medical Sciences & Peking Union Medical College, Beijing 100005, China;

[2] International Center of Microvascular Medicine, Chinese Academy of Medical Sciences, Beijing 100005, China;

[3] Diabetes Research Center, Chinese Academy of Medical Sciences, Beijing 100005, China;

[4] Laboratory of Electron Microscopy, Ultrastructural Pathology Center, Peking University First Hospital, Beijing 100034, China.

[#] These authors contributed equally to this study.

*** Correspondence:**

Mingming Liu, Institute of Microcirculation, Chinese Academy of Medical Sciences & Peking Union Medical College, No.5 Dong Dan Third Alley, Dongcheng District, Beijing 100005, China. E-mail address: mingmingliu@imc.pumc.edu.cn

Funding

This work was supported by the Beijing Municipal Natural Science Foundation (Grant No. 7252093).

Conflicts of Interest: The authors declare no conflict of interest

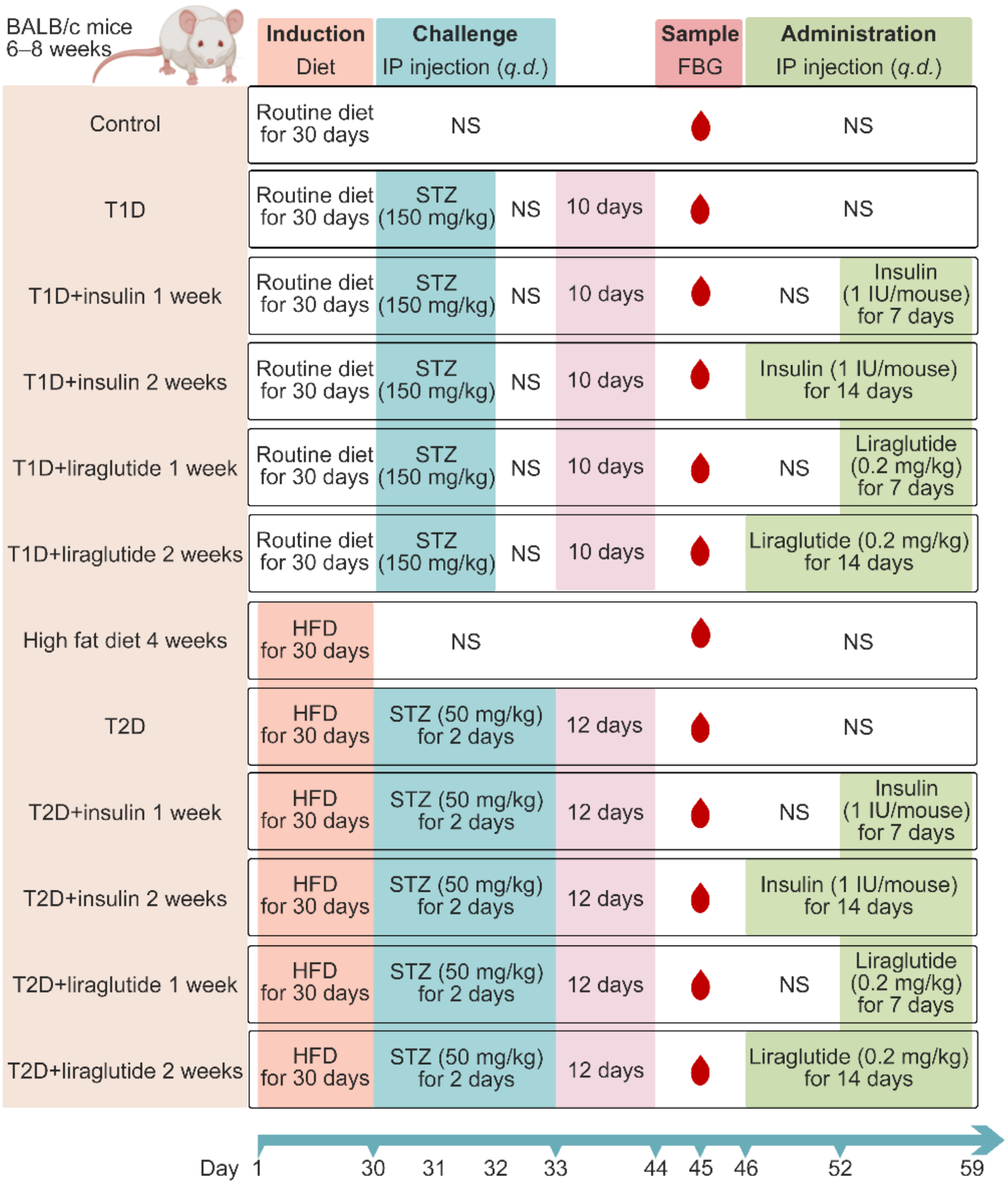

Supplementary figure 1 Schematic of the experimental design and timeline.

Supplementary Table 1 Summary of animal cohort allocation, attrition, and final sample sizes at each experimental endpoint

Experimental group	Initial enrollment (*n*)	Mortality / Attrition (*n*)	Final sample size (*n*)	Evaluated endpoint
Control	15	0	15	Baseline
HFD (4 Weeks)	15	0	15	Week 4 (Post-diet)
T1D (Untreated)	15	0	15	Baseline (Post-STZ)
T1D + Insulin	15	0	15	1 Week Post-treatment
T1D + Insulin	15	0	15	2 Weeks Post-treatment
T1D + Liraglutide	15	0	15	1 Week Post-treatment
T1D + Liraglutide	15	0	15	2 Weeks Post-treatment
T2D (Untreated)	15	0	15	Baseline (Post-STZ)
T2D + Insulin	15	0	15	1 Week Post-treatment
T2D + Insulin	15	0	15	2 Weeks Post-treatment
T2D + Liraglutide	15	0	15	1 Week Post-treatment
T2D + Liraglutide	15	0	15	2 Weeks Post-treatment
**Total**	180	0	180	-

Abbreviations: HFD, high-fat diet; T1D, type 1 diabetes; T2D, type 2 diabetes. All mice successfully met the disease induction criteria and survived until their respective predefined endpoints without adverse events requiring euthanasia prior to data acquisition.

Supplementary note: Analytical framework for multi-axial data

The dataset, by its multi-axial design, constitutes a third-order tensor, T, of dimensions 12 (disease/intervention states) × 6 (organs) × 10 (microcirculatory parameters). An element T_{ijk} represents the value of the kth parameter for the jth organ under the ith state. Our structure permits a multi-layered analytical approach, moving from elementary vector comparisons to complex system-level modeling. The section provides a formal framework for analyses that validating the dataset's internal consistency and demonstrating its utility for quantitative hypothesis testing (Figure 5, Supplementary figure 2).

1. Foundational analysis via data slicing and vectorial representation

The primary analytical strategy involves conditioning on two of the three axes to interrogate the distribution along the third. The reduces the tensor to a series of vectors, whose geometric and statistical properties can be quantitatively assessed, as it transforms a multi-modal dataset into a collection of discrete data points within a shared, high-dimensional vector space, making them amenable to geometric analysis (Figure 5).

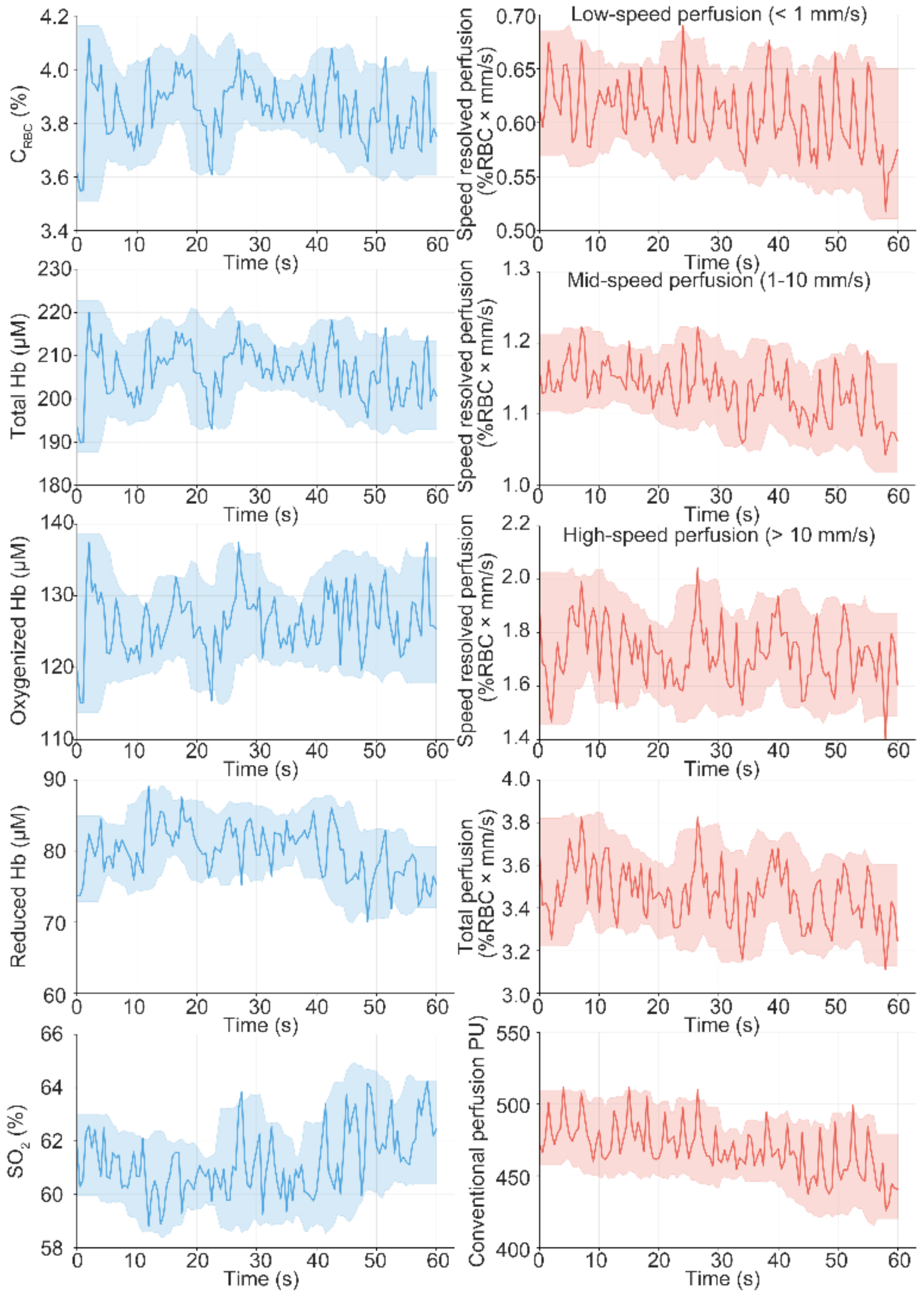

Supplementary figure 2 Representative 60-second trace of the 10-parameter microcirculatory physio-signature.

The Physio-signature vector V_{ij}

By fixing the disease state (*i*) and organ (*j*)[1], we obtain a 10-dimensional physio-signature vector, $V_{ij} = [T_{ij1}, T_{ij2}, ..., T_{ij10}]$, which represents the complete microcirculatory profile for that specific condition. Each vector V_{ij} constitutes a coordinate in a 10-dimensional Euclidean space, hereafter referred to as the physiological state-space. The origin or reference point in this space is naturally defined by the control vector, V_{1j}, representing the homeostatic baseline.

Quantifying state perturbation. The magnitude of deviation from a baseline (e.g., control, $i = 1$) can be quantified using the L^2-norm (Euclidean norm) of the difference vector. The overall physiological impact of a disease state i on organ j relative to control is:

$$Perturbation = \| V_{ij} - V_{1j} \|_2 = \sqrt{\sum_{k=1}^{10} (T_{ijk} - T_{1jk})^2}$$

The scalar metric provides a holistic measure of disease severity or therapeutic effect at the organ level.

Assessing mechanistic similarity. The cosine similarity metric can determine if a treatment restores not just the magnitude but the pattern of physiological parameters towards the healthy state. The metric moves beyond simple magnitude-based comparisons, for example, Euclidean distance, to assess the directional alignment of the state vectors within the 10-dimensional physiological space. The similarity between a treated state (e.g., T1D+Insulin, $i = 3$) and the control state ($i = 1$) in organ j is given by:

$$Sim(V_{3j}, V_{1j}) = \frac{V_{3j} \cdot V_{1j}}{\| V_{3j} \|_2 \| V_{1j} \|_2} = \frac{\sum_{k=1}^{10} T_{3jk} T_{1jk}}{\sqrt{\sum_{k=1}^{10} T_{3jk}^2} \sqrt{\sum_{k=1}^{10} T_{1jk}^2}}$$

The formula is derived directly from the geometric definition of the dot product in linear algebra, which relates the dot product of two vectors to the cosine of the angle θ between them:

$$V_{3j} \cdot V_{1j} = \| V_{3j} \|^2 \| V_{1j} \|^2 \cos(\theta)$$

By rearranging the foundational equation to solve for cos(θ), the similarity score is the cosine of the angle separating the treated-state and control-state vectors. The denominator, which is the product of the vectors' L^2-norms, serves to normalize the dot product, thereby isolating the directional component from the magnitude.

A value approaching 1 indicates that the angle θ between the two vectors is approaching 0°, implying that the treatment is restoring the relative proportions of the microcirculatory parameters towards the healthy profile. Conversely, a value approaching 0 signifies orthogonality ($\theta \approx 90°$), implying the pattern of the treated state is uncorrelated with the healthy state. A negative value would indicate that the vector is pointing in an opposing direction to the healthy profile.

2. Quantifying organ-specific and intervention-specific responses

The organ distribution vector U_{ik}

Fixing the disease state (i) and parameter (k) yields a 6-dimensional organ distribution vector, $U_{ik} = [T_{i1k}, T_{i2k}, ..., T_{i6k}]$. The vector profiles a specific parameter across all measured

organs. The vector profiles a specific parameter across all measured organs, representing a =shift in analytical perspective, instead of viewing the complete multi-parameter signature of a single organ, we are now examining the systemic footprint of a single parameter across the entire organ system.

Organ heterogeneity index. The variability of a parameter k across organs under state i can be quantified by the sample standard deviation of this vector's components:

$$S_{ik} = \sqrt{\frac{1}{6-1}\sum_{j=1}^{6}\left(T_{ijk} - \bar{T}_{ik}\right)^2}$$

where $\bar{T}_{ik} = \frac{1}{6}\sum_{j=1}^{6} T_{ijk}$ is the mean of T_{ijk} over all organs j. The formula measures the dispersion of data points around their mean. The term $T_{ijk} - \bar{T}_{ik}$ is the deviation of each organ's specific response from the systemic average. Squaring these deviations ensures that both positive and negative deviations contribute to the total variability and gives greater weight to larger deviations. The denominator (6−1) is used instead of 6 to provide an unbiased estimate of the population standard deviation from the sample data, namely Bessel's correction.

A large S_{ik} indicates high organ-specific heterogeneity in response to the disease or treatment. Conversely, a small S_{ik} indicates a low heterogeneity, suggesting a homogeneous or systemic response where parameter *k* is affected uniformly across all measured organs. Further analysis using hierarchical clustering on these vectors can reveal functional organ-clusters that respond similarly under specific pathological conditions.

The intervention response vector W_{jk}

Fixing the organ (*j*) and parameter (*k*) produces a 12-dimensional intervention response vector, $W_{jk} = [T_{1jk}, T_{2jk}, ..., T_{12,jk}]$, which traces the parameter's trajectory across all experimental groups. The vector provides a longitudinal view, allowing us to analyze how a single, specific physiological measurement behaves as a function of time or therapeutic condition, akin to a time-series analysis for a single biomarker.

Rate of therapeutic change. For time-course interventions (1 week vs. 2 weeks), we can model the rate of change. For instance, for insulin treatment in T1D mice, the average weekly rate of change (*b*) for parameter *k* in organ *j* can be estimated with a linear model, T(*t*) = a + bt, where *t* is time in weeks. The slope *b* is:

$$b = \frac{T_{T1D + Insulin\ 2w,j,k} - T_{T1D + Insulin\ 1w,j,k}}{2-1}$$

The formula is an application of the fundamental definition of a slope from algebra, the

change in the vertical axis divided by the change in the horizontal axis. The numerator represents the net change in the physiological parameter T over the observation period. The denominator represents the elapsed time, which in this case is one week. The resulting coefficient b has units of parameter units per week and represents the average rate of change under the core assumption of a linear response between the two time points.

Comparing these slope coefficients between different therapies (e.g., insulin vs. liraglutide) provides a quantitative basis for evaluating the pharmacodynamic velocity of each intervention. The magnitude of b indicates the speed of the therapeutic effect, for example, a larger absolute value means a faster-acting intervention, while the sign of b indicates the direction of change. Should more time points be available, this simple slope calculation could be extended to linear regression analysis to find the line of best fit, where b would be the regression coefficient.

3. System-level modeling and interaction effects

To transcend sliced analysis and capture the systemic nature of the data, multi-way models are essential.

Dissecting interaction effects with multi-way ANOVA

A critical question is whether treatment's effect is organ dependent. This is a question of statistical interaction. For any given parameter k, we can fit a two-way Analysis of Variance (ANOVA) model that partitions the total observed variance into components attributable to different sources or factors. The model is specified as:

$$Y_{ijk} = \mu_k + \alpha_i + \beta_j + (\alpha\beta)_{ij} + \varepsilon_{ijk}$$

where:

Y_{ijk} is the observed value of parameter k for the ith group and j^{th} organ.

μ_k is the grand mean for parameter k.

α_i is the main effect of the ith disease/intervention group, representing the average deviation from the grand mean caused by membership in group i.

β_j is the main effect of the jth organ, representing the average deviation from the grand mean attributable to organ type j.

$(\alpha\beta)_{ij}$ is the interaction effect between group and organ, which quantifies the extent to which the effect of group i is dependent on the level of factor j. A non-zero interaction term implies the effects of the intervention and organ are not additive.

ε_{ijk} is the random error term, which is assumed to be independently and identically drawn from a normal distribution with a mean of zero, i.e., $\varepsilon_{ijk} \sim N(0, \sigma 2)$

A statistically significant interaction term (p-value for $((\alpha\beta)_{ij} < 0.05)$ provides quantitative evidence that the effect of the disease or intervention (α_i) is not uniform across all organs but is significantly modulated by the organ type (β_j). It allows for the rejection of the null hypothesis

that there is no interaction (H_0: $(\alpha\beta)_{ij} = 0$ for all *i, j*). For a simultaneous test across all 10 parameters, a multivariate ANOVA (MANOVA) is the appropriate extension, as it can test the interaction hypothesis across all dependent variables at once while accounting for the covariance structure among them.

Reference

1. Kaiyala, K. J. Mathematical model for the contribution of Individual organs to non-zero y-intercepts in single and multi-compartment linear models of whole-body energy expenditure. *PLOS ONE*. **9**, e103301 (2014).

Supplementary figure legends

Supplementary figure 1. Schematic of the experimental design and timeline. Male BALB/c mice were randomized into a control group maintained on a standard chow diet, a cohort for T1D induction, and a cohort for modeling diet-induced insulin resistance and T2D. The T2D cohort was first fed a high-fat diet (HFD; 60% kcal from fat) for 4 weeks to establish a pre-diabetic, insulin-resistant state. Following the period, T2D was induced in these mice via two consecutive daily *i.p.* injections of low-dose STZ (50 mg/kg). Concurrently, the T1D cohort, maintained on standard chow, was induced via a single high-dose IP injection of STZ (150 mg/kg). The diabetic state was confirmed in T1D and T2D mice when FBG levels exceeded 200 mg/dL, measured 10 and 12 days post-STZ administration, respectively. Confirmed diabetic mice (both T1D and T2D) were then randomized into three subgroups for daily *i.p.* administration of vehicle (saline), insulin (1 IU), or the GLP-1 receptor agonist liraglutide (0.2 mg/kg). Microcirculatory data were acquired at two distinct therapeutic endpoints after 1 week and 2 weeks of continuous treatment. T1D, type 1 diabetes; T2D, type 2 diabetes; HFD, high-fat diet; *i.p.*, intraperitoneal; STZ, streptozotocin; FBG, fasting blood glucose.

Supplementary figure 2. Representative 60-second trace of the 10-parameter microcirculatory physio-signature. Raw data stream acquired from tissues illustrating the structure of the foundational data vector used. The recorded parameters are grouped by microcirculatory function. Left column (blue): The microcirculatory oxygenation profile, comprising the red blood cell tissue fraction (C_{RBC}), total hemoglobin (total Hb), oxygenated hemoglobin (oxygenized Hb), reduced hemoglobin (reduced Hb), and tissue oxygen saturation (SO_2). Right column (red): The microhemodynamic profile, consisting of total blood perfusion, speed resolved deconstruction into low-speed (<1 mm/s), mid-speed (1-10 mm/s), and high-speed (>10 mm/s) components, and the conventional perfusion signal (PU). Each 60 sec recording of this 10-dimensional vector constitutes a single dataset point for one organ within the complete

third-order data tensor. The shaded area represents the 95 % CI. C_{RBC}, red blood cell tissue fraction; SO_2, oxygen saturation; Hb, hemoglobin concentration; CI, confidence interval.
